# Identification and removal of laser-induced noise in photoacoustic imaging using singular value decomposition

**DOI:** 10.1364/BOE.8.000068

**Published:** 2016-12-05

**Authors:** Emma R. Hill, Wenfeng Xia, Matthew J. Clarkson, Adrien E. Desjardins

**Affiliations:** 1 Department of Medical Physics and Biomedical Engineering, University College London, Gower Street, London, WC1E 6BT, UK; 2 Translational Imaging Group (TIG), Centre for Medical Image Computing (CMIC), Dept. of Medical Physics and Biomedical Engineering, University College London, Gower Street, London, WC1E 6BT, UK; 3 Equal contribution

**Keywords:** (100.0100) Image processing, (110.4280) Noise in imaging systems, (110.5120) Photoacoustic imaging

## Abstract

Singular value decomposition (SVD) was used to identify and remove laser-induced noise in photoacoustic images acquired with a clinical ultrasound scanner. This noise, which was prominent in the radiofrequency data acquired in parallel from multiple transducer elements, was induced by the excitation light source. It was modelled by truncating the SVD matrices so that only the first few largest singular value components were retained, and subtracted prior to image reconstruction. The dependency of the signal amplitude and the number of the largest singular value components used for noise modeling was investigated for different photoacoustic source geometries. Validation was performed with simulated data and measured noise, and with photoacoustic images acquired from the human forearm and finger *in vivo* using L14-5/38 and L40-8/12 linear array clinical imaging probes. The use of only one singular value component was found to be sufficient to achieve near-complete removal of laser-induced noise from reconstructed images. This method has strong potential to increase image quality for a wide range of photoacoustic imaging systems with parallel data acquisition.

## 1. Introduction

Photoacoustic (PA) imaging couples the molecular contrast of optical absorption with the structural contrast and spatial resolution of ultrasound (US) imaging [1]. With this hybrid imaging modality, tissue is irradiated with pulsed or modulated excitation light that is scattered in tissue and absorbed by chromophores. Absorption is followed by rapid increases in local temperature, which lead to the generation of US waves that are received for image formation. There is growing interest in the use of PA imaging to guide clinical procedures [2–14]. Recent examples include the guidance of prostate biopsies [2], sentinel lymph node biopsies [3], breast biopsies [4], liver biopsies [5], prostate brachytherapy [6], nerve blocks [7,8], and fetal surgeries [9]. From the standpoint of clinical translation, the use of clinical US imaging probes for reception can be advantageous as it allows for inherently co-registered PA and B-mode US images. Dedicated hardware modules allow for parallel data acquisition from all transducer elements in an imaging probe [2–14].

As the attenuation of excitation light over cm-scale depths in tissue can be substantial, achieving high sensitivity to generated US tends to be of critical importance in PA/US imaging systems. The vulnerability of PA systems to external noise sources can limit sensitivity and degrade image quality. In the authors’ experience, noise produced by the electronics in certain commonly-used excitation light sources can be prominent; it is present in the US transducer signals with amplitudes comparable to those of PA signals.

Several methods have been considered for PA noise reduction. Averaging across consecutive PA images has the advantage of simplicity. However, as the image acquisition rate is limited by the repetition rate of the laser (typically in the vicinity of 10 to 200 Hz), achieving sufficient noise reduction and maintaining spatial resolution may not be compatible with tissue motion during imaging *in vivo*.

Here, singular value decomposition (SVD) was used to model and remove noise in PA images that derives from sources external to the ultrasound acquisition system. It was applied to each PA data matrix, which comprised radiofrequency (RF) data that were acquired in parallel from all transducer elements in the imaging probe immediately following one excitation light pulse. External noise, which was induced from the laser in the excitation light source (*cf*. Discussion), was assumed to be additive with respect to PA signals and it was modelled with a truncated sum of singular value components (SVCs). Denoising was performed by subtracting the modelled noise from the acquired data matrices. Whereas SVD has been used in previous studies to obtain a sparse representation of the signal [15–17], it was used here to obtain a sparse representation of the noise.

## 2. Materials and methods

### 2.1 Imaging system

PA excitation light was provided by an optical parametric oscillator (OPO) system (VersaScan L-532, GWU-Lasertechnik, Erftstadt, Germany) pumped by an Nd:YAG laser (repetition rate: 10 Hz; Quanta-Ray, INDI-40-10, Spectra-Physics, Santa Clara, CA, USA). The Nd:YAG laser was pumped with a flash lamp. Excitation light at a wavelength of 800 nm was delivered to tissue in dark-field mode via a randomized array of optical fibres, which provided a uniform rectangular beam pattern (~50 × 2 mm) at the tissue surface. The light fluence at the tissue surface was less than 10 mJ cm^−2^, which is below the maximum permissible exposure limit [18].

US detection was performed using a commercial US imaging system (SonixMDP, Analogic Ultrasound, Peabody, MA, USA) operated in its research mode. Two clinical linear array imaging probes with nominal bandwidths of 5 - 14 MHz and 8 - 40 MHz (L14-5/38 and L40-8/12, Vermon, Tours, France) were used. Pre-beamformed RF data from all the transducer elements were sampled at 40 MS/s by a 128-channel data acquisition system (SonixDAQ, Analogic Ultrasound) and transferred to a PC. The data in the first 1.7 µs (2.6 mm) was zeroed to remove noise that was present even in the absence of excitation light. Additionally, zero padding was performed to correct for a 4 µs acquisition trigger delay.

PA image reconstruction was performed using a Fast Fourier Transform method, implemented offline using the *k*-Wave MATLAB toolbox, with a uniform speed of sound (1540 m/s) [19]. Prior to reconstruction, the acquired RF data matrix was processed to identify and remove laser-induced noise, as detailed in the following section.

### 2.2 SVD denoising

Each acquired RF data matrix **X** was expressed in the form:

$$X=USV^{\text{T}}$$ (1)

using singular value decomposition (SVD). Here, **X** is a matrix of dimension n×p, where *n* is the number of samples acquired in each PA image from each transducer element, and *p* is the number of transducer elements. In the SVD decomposition, **U** and **V** are matrices of the left- and right-singular vectors, respectively, and **S** is the diagonal matrix of singular values that are ordered from largest to smallest. The left-singular vectors are an orthonormal basis for **X** that were used to provide a sparse representation for the noise. The RF data matrix was assume to comprise a sum of three terms: the PA signal **X_PA_**, the laser-induced noise **X_L_**, and a residual noise term ε:

$$X=X_{\mathrm{pa}}+X_{L}+\text{ε}.$$ (2)

An estimate of the laser induced noise, ${\tilde{X}}_{L}$ was obtained with a small number *k* of SVCs: ${\tilde{X}}_{L}=US_{L}V^{T}$, where **U** and **V** were identical to those in Eq. (1), and **S_L_** was formed by truncating **S**:

$$s_{\text{L}}\left(i\right)=\{\begin{array}{c} s\left(i\right),\;1\leq i\leq k\\ 0,i>k \end{array}$$ (3)

In Eq. (3), *s*_L_(*i*) and *s*(*i*) are the *i*^th^ diagonal elements of **S_L_** and **S**, respectively. The PA signal term and the laser-induced noise term could be separated in this way because of fundamental differences in their characteristics. With parallel data acquisition, the noise patterns were consistent across vectors in **X**, so that they had the appearance of planar wavefronts, whereas signals from a localized PA source comprised curved wavefronts. An estimate of the PA signal term ${\tilde{X}}_{PA}$ was obtained by subtracting the estimate of the laser-induced noise ${\tilde{X}}_{L}$: from the RF data matrix:

$${\tilde{X}}_{PA}=X-{\tilde{X}}_{L.}.$$ (4)

With the SVD denoising method presented here, a balance is sought: it is critical to accurately identify the noise whilst preserving the PA signal. It is therefore important to choose an appropriate number of SVCs for which the corresponding weights are zeroed for denoising. To obtain an indication of how this choice affects image quality, PA simulations were performed with *k*-Wave [19]. Two PA source geometries were simulated. In the first, there were four circular sources (1 mm diameter) with centres that spanned a depth range of 10 to 40 mm and a lateral range of 3 to 18 mm (0 mm corresponded to the lateral position of the first transducer element). In the second, there were five line sources that spanned an angular range of –31 to + 31 degrees and intersected centrally at a depth of 20 mm and a lateral distance of 20 mm. For both source geometries, a linear array of 128 point ultrasound detectors with flat frequency responses was used for signal detection. The medium had a uniform speed of sound (1540 m/s), and an absence of attenuation. Laser-induced noise from all transducer elements was obtained with the imaging system (L14-5/38 probe) when the excitation light source was on, but blocked so that the light did not reach the sample. This noise RF data, which corresponded to one excitation light pulse, was added to the PA RF data obtained from both source geometries. The maximum magnitude of the measured noise RF data was scaled to be 5 times as large as that of the simulated PA RF data to provide a realistic noise level that was encountered during *in vivo* experiments. For the simulation with four circular sources, SVD denoising was performed with different numbers of SVCs. For each number, the signal-to-noise ratio (SNR) was calculated. For SNR calculations, the signal for each circular source was defined as the mean pixel magnitude across a 2 × 2 mm square region enclosing the circular source; the noise, as the standard deviation across a spatial region similar to that spanned by the circular sources.

**Fig. 1:**
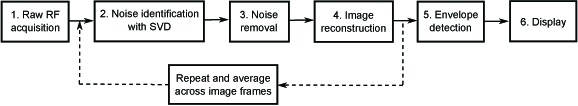
Steps performed to obtain a display of a denoised photoacoustic image. First, radiofrequency (RF) photoacoustic data from transducer elements of a linear-array US imaging probe were acquired in parallel (step 1). Laser-induced noise was identified using SVD (step 2) and then removed (step 3) prior to image reconstruction (step 4). Steps 1-4 were repeated when averaging over multiple PA acquisitions was required. Subsequently, envelope detection was performed with the Hilbert transform (step 5) and the resulting photoacoustic image was displayed on a logarithmic scale (step 6). For B-mode US imaging (steps not shown), acquisitions were performed using electronic focusing; the resulting images were inherently co-registered with the photoacoustic images since they were acquired with the same imaging probe.

*In vivo* imaging was performed on a forearm and a finger (palm side, between the first and second knuckle) of a healthy human volunteer. For forearm imaging, an agar gel block (2 cm in thickness) was positioned between the imaging probe and the skin surface, with ultrasonic coupling gel on both sides of the block. For finger imaging, water served as the coupling medium. In each case, B-mode pulse-echo US and PA images were acquired. The PA images were obtained with and without averaging across 31 consecutive frames, and with different numbers of SVCs used for denoising (Fig. 1

).

## 3. Results

**Fig. 2:**
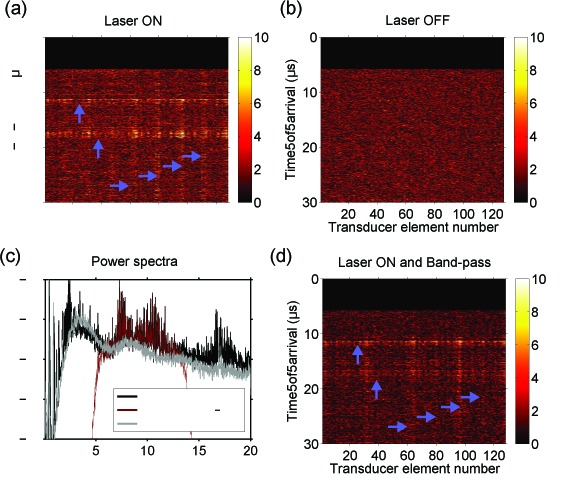
Radiofrequency (RF) data from transducer elements of the L14-5/38 US imaging probe. When the laser source was on but light was not delivered to tissue, laser-induced noise in the raw RF data manifested as horizontal and vertical bands (prominent instances pointed to with purple arrows) that varied across acquisitions (a). These bands were absent when the laser source was off (b). The power spectrum of the laser-induced noise, averaged across data from all transducer elements, overlapped with the nominal bandwidth of the transducer elements (c). Band-pass frequency filtering across the nominal bandwidth of the transducer elements (5 – 14 MHz) was insufficient to remove the laser-induced noise bands (d). In (a), (b), and (d), the absolute values of the RF data are displayed on a linear scale.

With the excitation light source turned on, laser-induced noise in the RF signals manifested as non-uniform horizontal and vertical bands [Fig. 2(a)

]. These noise bands varied with each acquisition; they were absent when the excitation light source was turned off [Fig. 2(b)]. The laser-induced noise was particularly prominent within the nominal bandwidth of the imaging probe [Fig. 2(c)]. As a result, it was still prominent after band-pass filtering across the imaging probe bandwidth (5th order Butterworth) [Fig. 2(d)].

**Fig. 3:**
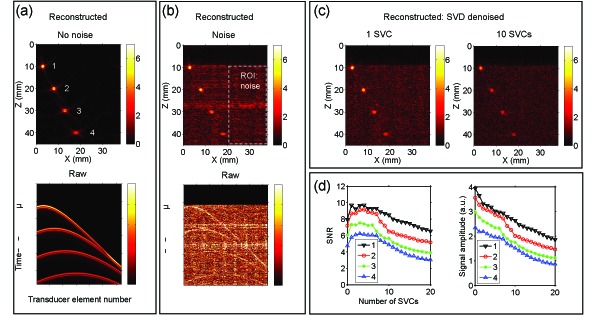
Laser-induced noise identification with singular value decomposition (SVD) using simulated signals from circular sources. Radiofrequency data for each transducer element originating from four circular sources at varying depths was simulated [(a), bottom]. Reconstructions were performed in the absence of laser-induced noise [(a), top] and in the presence of this noise [(b), top]. Laser-induced noise was experimentally acquired from the imaging probe with the laser on and with light not delivered to tissue, and added to the raw simulated data [(b), bottom]. Laser-induced noise identification with one singular value and subsequent denoising yielded a substantial improvement in photoacoustic image quality [(c), left]. When ten singular value components (SVCs) were used, the magnitudes of the signals originating from the circular sources were smaller relative to the background noise [(c), right]. The signal-to-noise ratio of the reconstructed images, with the signal for each circular source defined as the mean pixel magnitude across a 2 × 2 mm square region enclosing the circular source and the noise as the standard deviation across a spatial region indicated by the dashed box [(b), top], remained approximately constant when 1 to 7 SVCs were used and it decreased monotonically for larger numbers of SVCs [(d), left]. The peak signal magnitudes from the four sources decreased monotonically with the number of SVCs [(d), right]. The data in (a), (b), and (c) are plotted on linear scales.

In the simulated PA images obtained with circular sources, laser-induced noise was similar in magnitude to signals from the deepest source. The RF signals arising from these sources had characteristic curved waveforms that contrasted in shape with the horizontal and vertical noise bands [Figs. 3(a)-3(b)

]. Laser-induced noise identification with one SVC and subsequent denoising yielded a substantial improvement in PA image quality. When ten SVCs were used, the magnitudes of the signals originating from the circular sources were smaller [Fig. 3(c)]. The SNR remained approximately constant when 1 to 7 SVCs were used, and it decreased monotonically for larger numbers of SVCs. The peak signal magnitudes from the four sources decreased monotonically with the number of SVCs [Fig. 3(d)].

**Fig. 4:**
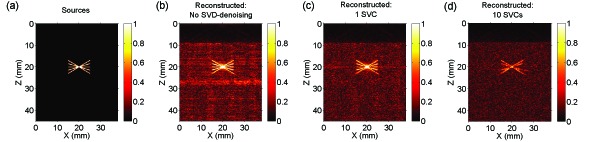
Laser-induced noise identification with singular value decomposition (SVD) using simulated signals from angled line sources. The five angled line sources, from which radiofrequency data for each transducer element was simulated, intersected at their centres (a). Experimentally-acquired laser-induced noise, which was the same as that used for simulations from circular sources (Fig. 3), manifested prominently in the reconstructed photoacoustic image (b). Laser-induced noise identification with one singular value component (SVC) and subsequent denoising yielded a substantial improvement in photoacoustic image quality overall (c). The signal from the horizontal line was apparent but its magnitude was smaller. When ten SVCs were used, the signal magnitudes from all lines relative to the background noise were smaller (d); the signal from the horizontal line was absent. All reconstructed photoacoustic images were normalised to their maximum values and displayed in the same linear scale (0-1).

As with the circular sources, laser-induced noise manifested prominently in the simulated PA images obtained with simulated angled line sources [Figs. 4(a)-4(b)

]. Laser-induced noise identification with one SVC and subsequent denoising yielded a substantial improvement in PA image quality overall. Signals from all lines were preserved by denoising; that from the horizontal line had a diminished magnitude and an artifact extending beyond this line at the same depth was induced [Fig. 4(c)]. When ten SVCs were used, the signal magnitudes from all lines were smaller; the signal from the horizontal line was absent but those from other lines were clearly apparent.

**Fig. 5:**
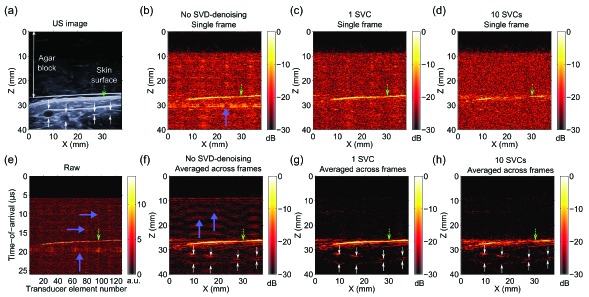
Laser-induced noise identification with singular value decomposition (SVD) in photoacoustic images acquired from a human forearm *in vivo.* These data, which were acquired with an L14-5/38 ultrasound (US) imaging probe, were co-registered with a B-mode pulse-echo US image (a). With B-mode US imaging, four superficial blood vessels were apparent (top and bottom of each vessel indicated with white arrows). Beneath the overlying agar block, there was a thin layer of US gel before the skin surface (dashed green arrow). Laser-induced noise manifested as prominent bands [(b), thick purple arrow] with a magnitude comparable to that of signals from the skin surface and larger than that of signals from blood vessels (not apparent). Laser-induced noise identification with 1 singular value component (SVC) and subsequent denoising yielded a substantial improvement in photoacoustic image quality, with the noise band absent (c). When 10 SVCs were used, the magnitude of the signal from the skin surface was reduced (d). In the raw radiofrequency data, vertical and horizontal noise bands were apparent [(e), prominent examples indicated with thick purple arrows]. When averaging across 31 PA images was performed, signals from the blood vessels were apparent but laser-induced noise across the image (prominent examples indicated with thick purple arrows) had comparable magnitudes (f). When averaging across PA images and SVD-denoising with 1 SVC were performed, the laser-induced noise was absent and signals from the blood vessels were clearly visible (g). The signals from the skin surface and the blood vessels were smaller when 10 SVCs were used (h). All reconstructed photoacoustic images were normalised to their maximum values and displayed on logarithmic scales with the same dynamic range (30 dB). The raw data in (e) is displayed on a linear scale.

The human forearm comprised anatomical features that were similar in geometry to the sources used in simulations: blood vessels that were approximately circular and a line-like skin surface. With B-mode US imaging, four superficial blood vessels were apparent [Fig. 5(a)

]. Laser-induced noise was similar in magnitude to PA signals from the skin surface and larger than that of signals from blood vessels [Fig. 5(b)]. Laser-induced noise identification with 1 SVC and subsequent denoising yielded a substantial improvement in PA image quality: the noise bands were absent [Fig. 5(c)]. When 10 SVCs were used, the magnitude of the signal from the skin surface was reduced [Fig. 5(d)]. In the raw RF data, vertical and horizontal noise bands were apparent [Fig. 5(e)]. Averaging across 31 PA images allowed for visualisation of blood vessels. Laser-induced noise remained after this averaging, however, and it was similar in magnitude to signals from the blood vessels [Fig. 5(f)]. When averaging across PA images and SVD-denoising with 1 SVC were performed, the laser-induced noise was absent and signals from the blood vessels were clearly visible [Fig. 5(g)]. The signals from the skin surface and the blood vessels were smaller relative to background noise when 10 SVCs were used [Fig. 5(h)].

**Fig. 6:**
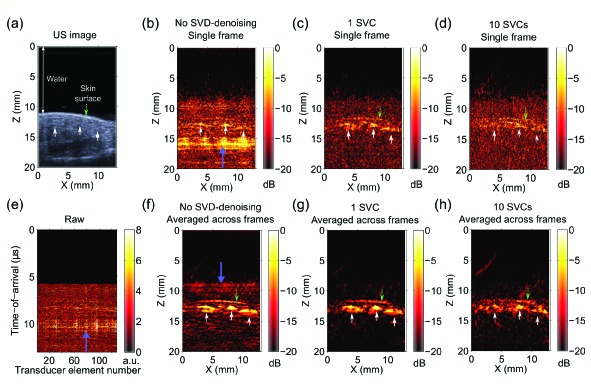
Laser-induced noise identification with singular value decomposition (SVD) in photoacoustic images acquired from a human finger *in vivo*. These data, which were acquired with an L40-8/12 high frequency (US) imaging probe, were co-registered with a B-mode pulse-echo US image (a). With B-mode US imaging, three superficial blood vessels (upward white arrows) were identified based on slight intensity variations over time (data not shown). Laser-induced noise manifested as prominent bands [(b), thick purple arrow] with a magnitude larger than that of signals from the blood vessels (upward white arrows). Laser-induced noise identification with 1 singular value component (SVC) and subsequent denoising yielded a substantial improvement in photoacoustic image quality, with the noise band absent and with signals from the skin surface present (c). When 10 SVCs were used, the magnitude of the signals from the skin surface and from the blood vessels was reduced relative to the background noise (d). In the raw radiofrequency data, vertical and horizontal noise bands were apparent [(e), prominent example indicated with a thick purple arrow]. When averaging across 31 PA images was performed, signals from the blood vessels were apparent but laser-induced noise across the image (prominent example indicated with a thick purple arrow) was present (f). When averaging across PA images and SVD-denoising with 1 SVC were performed, the laser-induced noise was absent and signals from the blood vessels and skin surface were clearly visible (g). The signals from the skin surface and the blood vessels were smaller relative to the background noise when 10 SVCs were used (h). All reconstructed photoacoustic images were normalised to their maximum values and displayed on logarithmic scales with the same dynamic range (20 dB). The raw data in (e) is displayed on a linear scale.

PA imaging of a human finger *in vivo* extended validation of the SVD denoising method to data acquired from a high frequency US imaging probe. With B-mode US imaging, three superficial blood vessels were identified based on slight intensity variations over time [Fig. 6(a)

]. Laser-induced noise manifested as prominent bands that were larger in magnitude larger than signals from the blood vessels [Fig. 6(b)]. Laser-induced noise identification with 1 SVD component and subsequent denoising yielded a substantial improvement in PA image quality, with an absence of noise bands and with signals from the skin surface present [Fig. 6(c)]. When 10 SVD components were used, the magnitude of the signals from the skin surface and from the blood vessels was reduced relative to the background noise [Fig. 6(d)]. In the RF data, vertical and horizontal noise bands were apparent [Fig. 6(e)]. When averaging across 31 PA images was performed, signals from the blood vessels were apparent but laser-induced noise across the image was present [Fig. 6(f)]. When averaging across PA images and SVD-denoising with 1 SVD component were performed, the laser-induced noise was visually absent and signals from the blood vessels and skin surface were clearly visible [Fig. 6(g)]. The signals from the skin surface and the blood vessels were smaller relative to the background noise when 10 SVD components were used [Fig. 6(h)].

## 4. Discussion and conclusion

Singular value decomposition was well suited to identifying and removing laser-induced noise that had similar manifestations in RF data from different transducer elements, in the context of a PA system with parallel detection. As a result of this similarity, laser-induced noise was well modelled with the first SVC. By comparison, PA signals from localised sources tend to give rise to curved wavefronts in the RF data, with temporal offsets that vary with the spatial position of the transducer element, so that they were poorly modelled with a small number of SVCs. Thus, zeroing the weight of the first SVC largely preserved the PA signals and removed the noise.

The denoising method presented here was validated with simulations and two different imaging probes *in vivo*. Simulations with circular sources demonstrated that the effectiveness of this method was insensitive to the source depth within the tested depth range of 40 mm. At much greater depths, the signal wavefronts in the RF data arising from circular sources are more planar, so that they may be more readily interpreted as laser-induced noise. In the simulations of purely horizontal line segments, the near-planarity of the signal wavefronts in the RF data resulted in prominent signal magnitude reductions in the reconstructed images after denoising. Therefore, one limitation of this method is the potential for deleterious effects on images of extended photoacoustic sources in tissue that are close to horizontal, such as skin surfaces.

A data-driven approach to identifying laser-induced noise, such as that provided by SVD, is well matched to the complexities of the noise induction process. In this study, it is likely that the short-duration voltage pulses provided to the flashlamp within the pump laser induce noise in the US signals. This induction process can be expected to depend on a multitude of factors, such as the spatial positions and shapes of electronic components that transmit RF data. As a result, variations in the characteristics of the laser-induced noise across different types of US imaging consoles and probes can be expected. With SVD, variations in the efficiency with which electromagnetic signals from the excitation light source are coupled into electronic cables that transmit data from different transducer elements are accounted for by the SVC weights. The relevance of the SVD denoising method presented here to different PA acquisition systems is likely to depend on the magnitude of the laser-induced noise. When the magnitude of the noise is lower than that of the PA signal, the first few SVCs may include prominent contributions from the PA signal, so that zeroing them could be detrimental to image quality. In this study, the noise was comparable in magnitude to the largest PA signals from tissue. As a result, the use of only one SVC was effective for denoising and preserving PA signal magnitudes. In general, the optimal choice for the number of SVCs used for denoising will depend on the imaging context.

The SVD-based method for identifying and reducing laser-induced noise is complementary to other noise and artifact reduction methods. As with wavelet denoising [15, 20], SVD could also be used to reduce residual noise, for instance by performing a second truncation of the singular value matrix in which diagonal elements of the singular value matrix that are below a certain threshold are zeroed [15–17]. Examples of artifact reduction methods that have recently shown promise include localised vibration tagging [11], short-lag spatial coherence weighting [12, 21,22], and synthetic aperture PA-guided focused US [13].

To the authors’ knowledge, this study is the first to use SVD to identify and remove laser-induced noise. It has strong potential to increase image quality for a wide range of PA imaging systems with parallel data acquisition.
